# The impact of COVID-19 pandemic on sarcopenic obesity among children between 6 and 10 years of age: a prospective study

**DOI:** 10.1007/s00431-025-06067-y

**Published:** 2025-03-14

**Authors:** Bahar Öztelcan Gündüz, Aysu Duyan Çamurdan, Mücahit Yıldız, Fatma Nur Baran Aksakal, Emine Nükhet Ünsal

**Affiliations:** 1 General Pediatrics, Gülhane Training and Research Hospital, Ankara, Turkey; 2 Social Pediatrics, Medical Faculty, Gazi University, Ankara, Turkey; 3 Department of Public Health, Medical Faculty, Gazi University, Ankara, Turkey; 4 Department of Nutrition and Dietetics, Gülhane Training and Research Hospital, Ankara, Turkey

**Keywords:** Sarcopenic obesity (SO), COVID-19 pandemic, Children’s body composition, Physical activity, Dietary habits, Sleep patterns

## Abstract

This study aims to examine the effects of physical activity, eating habits, sleep patterns, and media use on children’s body composition during the COVID-19 pandemic, as well as the relationship of these factors with sarcopenic obesity (SO). This prospective cross-sectional study has involved 431 healthy male and female child participants aged between 6 and 10 years during the COVID-19 pandemic lockdown period. The daily routines of participants, including their dietary habits, levels of physical activity, and media usage patterns, have been assessed. The anthropometric measurements taken included body weight, height, body mass index (BMI), and skinfold thickness assessments. Body composition analyses have been conducted using the bioelectrical impedance (BIA) method to determine the total body fat and muscle mass as well as the fat percentage. It has identified obesity in 25.2% and SO in 9.5%. Children with SO have had mean BMI SDS of 2.67 ± 0.4 and mean waist circumference of 78.5 ± 9 cm. Fruit consumption OR = 2.68, 95% CI (1.13–6.31), the number of household members OR = 0.54, 95% CI (0.35–0.84), the duration of sitting time OR = 1.17, 95% CI (1.02–1.36)], and junk food consumption OR = 1.27, 95% CI (1.03–1.57)] have been found to be effective in the development of SO. *Conclusion*: The COVID-19 pandemic has had a significant impact on the body composition of children, resulting in an increased prevalence of obesity and sarcopenic obesity. This research highlights the critical importance of engaging in regular physical activity, consuming a balanced diet, and obtaining sufficient sleep, particularly during times of crisis.

**Table:** 

**What is Known:**
• *Sarcopenic obesity is a complex metabolic condition characterized by reduced muscle mass and increased adipose tissue.*
• *COVID-19 pandemic-related physical inactivity potentially has led to adverse effects on muscle mass composition.*
**What is New:**
• *First comprehensive assessment of sarcopenic obesity development in children during the COVID-19 pandemic, utilizing advanced bioelectrical impedance analysis (BIA) to evaluate changes in muscle mass and adipose tissue.*
• *Systematic evaluation of the impact of sedentary lifestyle and dietary habits on sarcopenic obesity during the unprecedented lockdown period.*

## Introduction

Sarcopenic obesity (SO) is an emerging concern in pediatric populations because of the dual epidemics of obesity and physical inactivity. It is characterized by the coexistence of obesity and sarcopenia (loss of muscle mass and strength). It is increasingly recognized as a significant health concern, even among children. Although it was previously thought to primarily affect the elderly, the recent research suggests that SO also affects the pediatric population [1]. In other words, emerging evidence suggests that SO can also manifest in younger populations, including children, particularly in the context of rising obesity rates and sedentary lifestyle. As a result, as the research shows the prevalence of SO in children and adolescents range from 5.7 to 69.7% in girls and from 7.2 to 81.3% in boys [2].

Moreover, childhood obesity is a growing global health problem. It has been estimated that 254 million children and adolescents are expected to become obese by 2030 [3, 4]. In Turkey, the prevalence of overweight and obesity among children aged 6 to 9 years was reported to be 14.3% and 6.5%, respectively [4].

Obesity and SO are closely associated with metabolic syndrome and cardiometabolic health in children [5], and they can lead to various complications as orthopedic problems, liver steatosis, and musculoskeletal disorders [6]. In addition, sarcopenic obesity in children can have long-term health consequences, including an increased risk of chronic diseases as type 2 diabetes, cardiovascular disease, and certain cancers [7].

However, few studies have investigated the impact of dietary habits, physical activity, sleep patterns, and media use on SO development in children during the COVID-19 pandemic. Yet, a comprehensive examination of these factors and their relationship with SO in children is necessary to address this growing public health concern. By understanding the unique challenges of the pandemic and their impact on childhood SO, evidence-based interventions and policies can be developed to mitigate the long-term health consequences of COVID-19.

As a result, the objective of this study has been to conduct a comprehensive investigation of the effects of physical activity, dietary habits, sleep patterns, and media use on body composition in children during the COVID-19 pandemic and to explore their relationship with SO. The findings of this study can inform the development of targeted strategies to prevent and manage SO in children which will ultimately improve their health outcomes and quality of life.

## Methods

### Study design and population

**Fig. 1:**
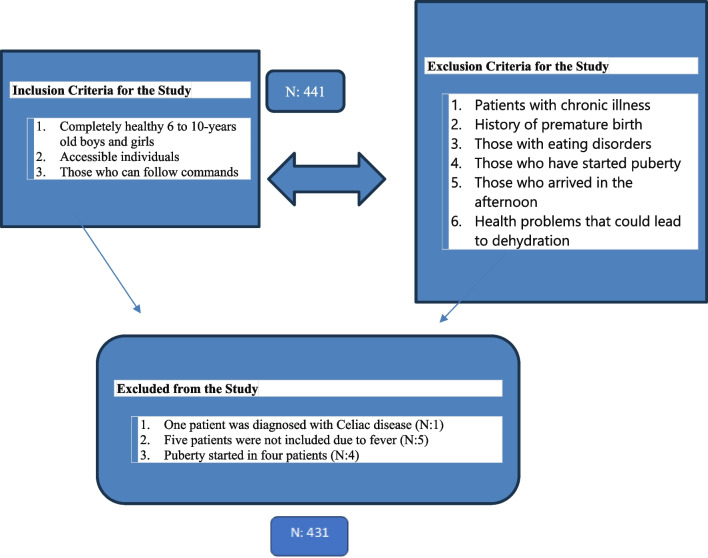
Inclusion and exclusion criteria

This prospective cross-sectional study consisted of male and female participants between 6 and 10 years old who attended the General Pediatrics Outpatient Clinic at Gulhane Training and Research Hospital, a tertiary hospital in Ankara, for routine checkups between December 16, 2021, and March 16, 2022, during the COVID-19 pandemic lockdown period. The study was approved by the Scientific Research Ethics Committee of Gulhane Training and Research Hospital (Approval No: 2021–350), and it included 431 children were initially identified as potentially eligible (Fig. 1).

In order to define the sample for this study, a physical activity questionnaire was conducted over a 1-week duration to assess the activity levels of children aged 6 to 10 years presenting at the clinic. The questionnaire revealed a physical activity prevalence of 38%. Based on this prevalence, a minimum sample size of 354 participants was calculated using a 95% confidence interval, a 5% margin of error, and an 80% power. To ensure equate representation of active individuals within the sample, the study employed sequential analysis to evaluate whether sufficient sample sizes were achieved for both active and inactive groups after the initiation of the study. Subsequent sequential data analysis was conducted to identify differences between groups, confirming the sample size’s adequacy, leading to the inclusion of 441 children in the study. Of these, one child was excluded due to a diagnosis of celiac disease, five due to dehydration during febrile episodes, and four due to the onset of puberty. These children were referred to appropriate clinics for continued management of their conditions. Consequently, the study was completed with a total of 431 healthy children. Based on the evaluations, sufficient sample sizes were reached for both the active and inactive groups. Given the lack of previous publications on this topic and the uncertainty regarding physical activity prevalence, this methodology was chosen, aiming for a participant loss of less than 20%.

During the study period, a previously developed physical activity (PA) questionnaire was used to determine whether completely healthy children aged 6–10 years were physically active or inactive. This form is a Turkish adaptation of the physical activity questionnaire for older children (PAQ-C) developed by Kowalski, and its validity and reliability were tested by Sert et al. [8, 9]. It inquired about the activities performed in the last 7 days (jump rope, soccer, basketball, gymnastics, etc.), participation in physical education classes, activities during recess, lunch time, after school, evenings, and weekends and also the frequency of leisure activities in the last 7 days.

Furthermore, the study also examined the frequency of sports, games, dance, and other physical activities across all 7 days of the week. The form also asks about leisure-time activities (sports, dance, and playing games) performed in the last 7 days. The frequency of behaviors was calculated using a five-point Likert scale (never = 1 point, 1–2 times = 2 points, 3–4 times = 3 points, 5–6 times = 4 points, and 7 or more times = 5 points). The first item lists 21 activities. For example, if a person performed the first item 3–4 times, their score would be 21 × 3 = 63, which is then divided by the number of activities in the first item (21) to obtain the average. The ninth item represents all 7 days of the week.

The children in the study were asked about the frequency with which they engaged in sports, games, dance, or other physical activities during the previous week and were requested to fill out the form for each day of the week. The score for the ninth item was divided by 7 (number of days) to calculate the average score. The total score was derived from the responses to the nine items in the questionnaire. The minimum score for each item in the PAQ-C was 1, and the maximum score was 5. The minimum total score on the PAQ-C was 9, and the maximum score was 45. The 10th PAQ-C item was not included in the scoring. If a child had any condition that prevented them from engaging in physical activity during that week, the PAQ-C was not evaluated.

As far as reliability is concerned, the test–retest reliability was at an acceptable level in the original scale (*r* = 0.75 for males, *r* = 0.82 for females). Additionally, the answering time of the form was determined as 20 min following pre-treatment [8, 9]. Although a clear classification could not be made in the original questionnaire, Sert et al. reported that a score of 9–14 could be classified as inactive (sedentary), a score of 15–24 as low-level PA, a score of 25–34 as moderately active PA, and a score of 35–45 as active.

### Dietary and lifestyle data collection

The questionnaire prepared for the study group comprised of 78 questions. The first 24 parameters gathered demographic information about the participants (age, sex, birth weight, gestational age, parents’ ages, education, whether they received breastmilk, and if so, for how long), and these were recorded in the form. Questions 25–50 covered family type (nuclear, extended, divorced family), mode of transportation to school (walking, by school bus or car), approximate distance of the school for those walking (the distance from home to school was estimated based on parental reports), participants’ meal patterns during the day (breakfast, lunch, and dinner), wake-up and bedtimes, total daily screen time (video, mobile phone, tablet, and television evaluated separately), and whether the screen was used for educational purposes, games, etc., which were also recorded separately.

The participants were asked to record their daily step count from the time they woke up until they went to bed using a pedometer or smartphone application. That is, they were asked to record their step counts for 3 days, and the average was recorded. In addition, the duration of sitting in front of a television or screen without any physical activity was obtained from the parents and it was also recorded.

In the study, junk food is defined as food high in calories but low in nutritional value. To assess the consumption of junk food, the families were asked to maintain a food diary. In the food diary, the participants were requested to record their consumption of food (junk foods, fruits, and meals), junk food (chocolate, crackers, chips, fruit juices, sodas, instant noodles, potato chips, sugar-containing foods, fast foods, ready-made cakes, pastries, cookies, ice cream, breakfast cereals with high sugar and low fiber content, pizza, etc.), and fruit intake. The food diaries kept by the parents were then reviewed and the quantities were recorded. The portion sizes for fruit were also recorded using measurements determined by the Turkish Dietary Guidelines (TUBER) [10] for children aged 7–10 years. Moreover, as another step of the study, nutrition-related data were recorded through a 24-h retrospective food consumption record, including the foods and beverages consumed by the children and the amounts of ingredients added during preparation. Based on this information, the daily energy and macro–micronutrient intake status of the participants were examined and analyzed using the Nutritional Information Systems Package Program (BEBIS 8.2) [11].

### Anthropometric and body composition measurements

The measurements were conducted by the same individual, an experienced pediatric health specialist and pediatric dietitian, utilizing standardized tools to ensure consistency and accuracy. Firstly, the height measurements were taken using a stadiometer (SECA 767, Hamburg, Germany) sensitive to 0.1 cm, with the participant standing upright, head straight, and looking straight ahead without shoes. Secondly, the weight measurements were also performed using a scale (SECA 767, Hamburg, Germany) with the children in their undergarments [12, 13].

As well as these, the waist circumference was measured using a nonstretchable tape in the standing position at the end of expiration and at the midpoint between the lowest rib and iliac crest. Right and left midupper arm circumferences (MUAC) were measured using nonstretchable but flexible tape.

Thirdly, the dominant and nondominant hands of the participants were recorded. Fourthly, the skinfold thickness measurements (Holtain Skinfold Caliper, Made in Britain) were performed by pinching the skin at predetermined points, approximately 1 cm away from the measurement site by following the guidelines in the literature which ensured that no muscle tissue was included between the thumb and index finger. The fingers were maintained at the same pressure on the calipers until the measurement was completed.

Once the anthropometric measurements were completed, the participants’ total body muscle and fat ratios (in kilograms), fat percentage (%), fat and muscle volumes, and torso muscle and fat (in kg) were measured using a Tanita BC 418 device (TANITA Corporation, Tokyo, Japan). Additionally, grip strength was measured in both hands using a Camry EH101 (Camry Corporation, Hong Kong, China). In line with this, the participant was first instructed on what to do, and after this, the dynamometer needle was zeroed. The participant was then asked to squeeze the grip of the device with full strength without touching the body. This process was repeated three times. The average value of observed on the needle was recorded in kilograms. The grip strength was recorded in the form of dominant hand strength and the highest measured grip strength.

### Evaluation of parameters

During the study, anthropometric measurements were recorded, including body weight (kg), height (cm), and body mass index (BMI) kg/m^2^) by one of the researchers. It should be noted that the World Health Organization (WHO) defines children above the 85th percentile of BMI for age and sex as overweight. Therefore, children above the 95th percentile of BMI according to age and sex were considered obese by WHO [3].

In addition to define which participants were obese, subcutaneous fat tissue was also measured using a caliper with the value indicated on the needle (cm). The Tanita device was used to measure the impedance (800 μA; 50 kHz) of the entire body and limbs separately in kilograms and percentages, but the grip strength was recorded in kilograms (kg).

### Method for defining sarcopenic obesity

When one looks at the literature, it can be seen that McCarty [14] and Kim [5] have defined sarcopenic obesity. In this method, BMI *z* scores were divided into five separate ranges according to age and sex. The muscle fat ratio (MFR) was calculated by dividing the skeletal (appendicular) muscle mass by the total body fat mass. The sum of the muscle mass of the arms and legs was utilized to determine the appendicular muscle mass.

The average and standard deviation of the obtained MFR were then calculated for each BMI *z* score quintile. MFR equal to the average minus 2 standard deviations was found for the middle quintile of the BMI *z* score. SO was determined to have MFR value below this threshold and to be in the highest quintile according to the BMI *z* score. The researchers checked whether all participants fell into the correct percentile ranges according to Turkish children’s growth curves and into the appropriate quintiles according to their BMI *z* scores [15].

### Statistical analysis

Statistical analyses were performed using the SPSS software (IBM SPSS Statistics for Windows, Version 22.0, Armonk, NY, USA), and the conformity of continuous variables to a normal distribution was examined using the Kolmogorov–Smirnov test. In the analysis, descriptive statistics are presented as mean ± standard deviation for normally distributed continuous variables and/or median (min–max) and percentiles for non-normally distributed variables whereas categorical data are expressed as numbers and percentages.

Firstly, descriptive statistics were calculated for all groups. Secondly, the categorical data for the group defined with sarcopenic obesity (SO) using the McCarty et al. [14] method were analyzed using the chi-square test, and continuous data were evaluated using the Mann–Whitney *U* test. Both univariate and multivariate analyses were conducted. For the multivariate analysis, the possible factors identified by the univariate analyses were further entered into the logistic regression analysis to determine independent predictors of patient outcomes. Moreover, Hosmer–Lemeshow goodness-of-fit statistics were used to assess model fit. A 5% type-I error level was used to determine statistical significance.

## Results

### General characteristics of the participants

Overall, the median age of the participants was 8 years (6–10). Two hundred thirty of the participants (53.4%) were female, and 201 of them (46.6%) were male. Overweight and obesity were found in 51 (11.8%) and 109 (25.2%) participants, respectively (Table 1).

**Table 1:** General characteristics of participants

Variable	*N*	%
Gender		
Girls	230	53.4
Boys	201	46.6
Overweight	51	11.8
Obese	109	25.2
Mother’s education (*n* = 431)		
Primary school	162	37.6
High school	140	32.5
University	129	29.9
Father’s education (*n* = 431)		
Primary school	134	31.1
High school	167	38.7
University	130	30.2
Family structure		
Nuclear family	390	90.5
Extended family	27	6.3
Divorced family	14	3.2
Transportation to school		
Walking	300	69.6
Vehicle/service	131	30.4
Parental perception of child’s weight		
Thin	152	35.3
Overweight	147	34.1
Obese	132	30.6
Breakfast meal		
Consumed	369	85.6
Skipped	62	14.4
Lunch meal		
Consumed	358	83.1
Skipped	73	16.9
Dinner meal		
Consumed	425	98.6
Skipped	6	1.4
Fruit consumption (portions)		
≤ 2	289	67.1
≥ 3	92	21.4
Junk food (a day)		
None	29	6.7
1 piece	219	50.8
2 pieces	77	17.9
≥ 3 pieces	106	24.6
	Mean ± SD	Median (min–max)
Age (*n* = 431) (years)	8.2 ± 1.3	8 (6–10)
Birth weight (*n* = 431) (g)	3258 ± 625	3250 (2950–3600)
Gestational age (weeks) (*n* = 431)	38.5 ± 1.8	38 (38–40)
Breastfeeding duration (months) (*n* = 421)	18.1 ± 10.3	18 (9–24)
Home-school distance (*n* = 300) (m)	398 ± 249	400 (200–500)
Sleep duration (hours) (*n* = 431)	9.5 ± 1.16	10 (6–12.5)
Online education duration (hours) (*n* = 290)	4.9 ± 1.4	5 (0.5–8)
Screen time (hours/day) (*n* = 431)	6.9 ± 2.8	7 (1–14)
Sitting time (hours/day) (*n* = 431)	6.8 ± 2.5	7 (1–14)
Step count (pedometer/day) (*n* = 431)	4777 ± 2384	4392 (1000–13,033)
Physical activity score (*n* = 431)	26.4 ± 5.6	26 (11.3–42)
Total energy intake (kcal/day) (*n* = 193)	1481 ± 332	1451 (763–2405)
Protein intake (E%) (*n* = 193)	15.3 ± 4	15 (7–28)
Carbohydrate intake (E%) (*n* = 193)	48.2 ± 10.1	47 (26–76)
Fat intake (E%) (*n* = 193)	36.4 ± 9.5	36 (12–59)

In total, 129 (29.9%) mothers and 130 (30.2%) fathers were university graduates. Three hundred ninety (90.5%) of the children were from nuclear families. Three hundred of them (69.6%) walked from home to school. The average home school distance was 398 ± 249 m. A total of 152 (35.3%) parents considered their children to be underweight. A total of 369 (85.6%) participants reported regularly consuming breakfast, 358 (83.1%) reported having lunch, and 425 (98.6%) reported eating dinner. Ninety-two (21.4%) participants reported consuming three or more portions of fruit per day and 106 (24.6%) reported consuming three or more servings of junk food per day (Table 1).

**Fig. 2:**
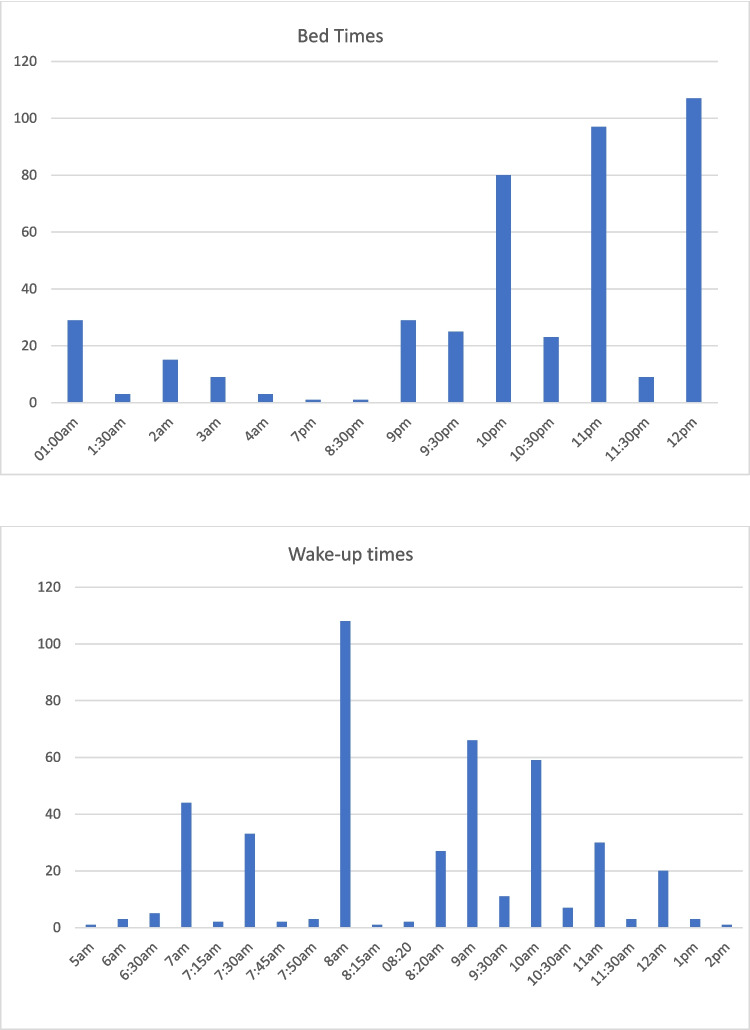
Bedtime and wake-up times of the participants

Additionally, the online lesson duration was 4.9 ± 1.4 h, total screen time was 6.9 ± 2.8 h, sitting time was 6.8 ± 2.5 h, the step count was 4777 ± 2384 steps. Moreover, the average physical activity score was 26.4 ± 5.6, total daily energy intake was 1481 ± 332 kcal/day, the percentage of daily protein intake was 15.3 ± 4%, that of carbohydrates was 48.2 ± 10.1%, and finally the fat percentage 36.4 ± 9.5% (Table 1). The participants’ average total daily sleep duration was 9.5 ± 1.16 h. Participants’ bedtime and wake-up times evaluations are shown in Fig. 2.

### Anthropometric measurements of participants

The anthropometric and body composition measurements of all participants according to age and sex are presented in Table 2. Sarcopenic obesity was observed in 41 participants (9.5%). As far as the sex of the participants are concerned, sarcopenic obesity was observed in 20 girls (48.8%) and 21 boys (51.2%). Age and sex were not different between patients with and without sarcopenic obesity (*p* = 0.64 and *p* = 0.53, respectively) (Table 3).

**Table 2:** Evaluation of children’s anthropometric measurements according to age and gender

Age (year)	Gender	Weight *z* score	Height *z* score	BMI *z* score	Waist circumference (cm)	Dominant arm, midarm circumference (cm)	Skapula skinfold thickness	Triceps skinfold thickness
6	Girl	− 0.68 ± 0.49	− 0.92 ± 0.53	− 0.44 ± 0.67	53.2 ± 6.4	17 ± 1.9	6.4 ± 4.5	9.7 ± 4.1
	Boy	− 0.15 ± 0.87	− 0.69 ± 0.71	0.25 ± 1.07	57.9 ± 8.7	19.1 ± 3.1	9.2 ± 7.3	11.4 ± 6.3
7	Girl	− 1.14 ± 0.95	− 0.33 ± 0.84	− 0.05 ± 1.03	57.1 ± 8.4	18.8 ± 2.7	9.6 ± 6.7	11.4 ± 5.5
	Boy	− 0.17 ± 1	− 0.08 ± 1.05	− 0.23 ± 0.91	57.6 ± 9.8	18.6 ± 3	8.2 ± 7.7	10.2 ± 6.0
8	Girl	0.43 ± 1	0.68 ± 0.77	0.25 ± 1.03	59.9 ± 8.5	20.3 ± 3.3	12.2 ± 8.7	13.7 ± 6.5
	Boy	0.20 ± 1.03	0.39 ± 0.89	0.04 ± 1	59.9 ± 9.5	19.4 ± 3.2	9.2 ± 8.3	10.5 ± 6.7
9	Girl	− 0.18 ± 0.80	− 0.32 ± 0.85	− 0.10 ± 0.84	60.1 ± 9.4	20.5 ± 3.5	11.7 ± 8.2	13.4 ± 6.0
	Boy	− 1.14 ± 1.02	− 0.31 ± 0.97	− 0.05 ± 1.04	66.3 ± 12.9	21.5 ± 4.3	14.2 ± 11.4	14.7 ± 8.4
10	Girl	0.19 ± 1.14	0.33 ± 1.03	0.10 ± 1.13	64.7 ± 13.5	21.3 ± 4.3	13.8 ± 10.2	14.5 ± 8.1
	Boy	0.15 ± 0.96	0.33 ± 0.92	0.06 ± 0.96	69.7 ± 12.5	22.7 ± 4.3	14.8 ± 10.8	15.4 ± 8.7
Age (year)	Gender	Appendicular muscle mass (kg)*(kg)	Fat mass (kg)	Muscle mass (kg)	MFR**	Dominant hand grip strength (kg)	Dominant hand grip strength/BMI	Waist skinfold thickness
6	Girl	6 ± 1.0	4.9 ± 1.6	17.1 ± 2.1	3.6 ± 0.7	8.3 ± 1.8	0.53 ± 0.11	5 ± 4.2
	Boy	7.1 ± 1.9	6.5 ± 3.4	20.1 ± 3.5	3.6 ± 1.1	9.7 ± 2.2	0.53 ± 0.12	7.1 ± 5.9
7	Girl	6.9 ± 1.8	6.6 ± 3.6	19.3 ± 4.0	3.3 ± 1	9.2 ± 2.2	0.55 ± 0.14	7.2 ± 5.5
	Boy	7.1 ± 2.2	6.2 ± 3.9	20.0 ± 4.4	3.8 ± 1.2	10.5 ± 2.6	0.64 ± 0.14	6.7 ± 3.1
8	Girl	8.5 ± 1.7	8.1 ± 3.7	22.8 ± 4.1	3.1 ± 0.9	12 ± 2.7	0.68 ± 0.16	9.3 ± 7.3
	Boy	8.3 ± 1.3	5.5 ± 4.2	22.0 ± 3.9	3.6 ± 1.1	12.2 ± 2.5	0.70 ± 0.13	6.8 ± 6.5
9	Girl	9.1 ± 2.2	8.6 ± 4.0	23.7 ± 4.9	3 ± 0.8	12.8 ± 3.6	0.72 ± 0.25	8.9 ± 6.7
	Boy	10.7 ± 3.3	10.7 ± 7.6	26.6 ± 5.5	3.3 ± 1.4	14.1 ± 2.9	0.73 ± 0.16	10.9 ± 9.4
10	Girl	10.3 ± 2.8	10.4 ± 6.6	26.3 ± 5.8	3.1 ± 1.1	14.3 ± 2.7	0.77 ± 0.17	10.9 ± 9.6
	Boy	12.1 ± 3.3	11.5 ± 6.6	29.6 ± 5.8	3.4 ± 1.6	16.3 ± 3.8	0.82 ± 0.22	12.2 ± 9.1

*Appendicular muscle mass: sum of the muscle mass of the right arm, left arm, right leg, and left leg

**Muscle/fat ratio

**Table 3:** Sample characteristics with and without sarcopenic obesity

	Total	Sarcopenic obesity	Without sarcopenic obesity	*P*
*N* (%)	431 (100)	41 (9.5)	390 (90.5)	
Gender				
Boys	201 (46.6)	21 (51.2)	180 (46.2)	0.53
Girls	230 (53.4)	20 (48.8)	210 (53.8)	
Age (years)	8.2 ± 1.3	8.2 ± 1.3	8.2 ± 1.3	0.64
BMI (kg/m^2^)	18.3 ± 4.2	25.5 ± 3.0	17.5 ± 3.6	0.001
BMI SDS	0.4 ± 1.4	2.6 ± 0.4	0.2 ± 1.3	0.001
Waist circumference (cm)	61.2 ± 11.2	78.5 ± 9	59.4 ± 9.8	0.001
Waist circumference caliper	8.8 ± 7.8	22.4 ± 7.1	7.4 ± 6.4	0.001
MUAC	20.2 ± 3.7	25.6 ± 2.3	19.6 ± 3.4	0.001
MUAC caliper	12.8 ± 7.1	23.8 ± 5.3	11.6 ± 6.2	0.001
Scapula caliper	11.4 ± 9.1	26.9 ± 5.4	9.7 ± 7.8	0.001
Body composition				
Muscle mass (kg)	23.3 ± 5.8	29.1 ± 4.6	22.7 ± 5.6	0.001
Fat mass (kg)	8.4 ± 5.3	17.5 ± 5.7	7.4 ± 4.3	0.001
MFR	3.3 ± 1.1	1.7 ± 0.3	3.5 ± 1	0.001
Hand grip	12.7 ± 3.5	14.4 ± 3.1	12.5 ± 3.5	0.001
Grip/BMI	0.7 ± 0.1	0.56 ± 0.11	0.72 ± 0.19	0.001
SMMa*	8.9 ± 2.9	11.9 ± 2.6	8.6 ± 2.8	0.001
SMMa/FM	1.2 ± 0.3	0.7 ± 0.1	1.3 ± 0.3	0.001
Total energy intake(kcal/day) (*n* = 193)	1481 ± 332	1444 ± 329	1485 ± 329	0.55
Macronutrients intake				
Protein (*n* = 193) intake (%E)	15.3 ± 4	16.1 ± 3.9	15.2 ± 4	0.30
Carbohydrate intake (%E) (*n* = 193)	48.2 ± 10.1	45.4 ± 9.3	48.5 ± 10.2	0.19
Fat intake (%E) (*n* = 193)	36.4 ± 9.5	38.4 ± 8.7	36.2 ± 9.5	0.36
Physical (*n* = 431) activity score	26.4 ± 5.6	25.7 ± 4.9	26.4 ± 5.7	0.58
Lifestyle changes (*n* = 431)				
Sleep duration (hours/day)	9.5 ± 1.16	9 ± 1.8	9.6 ± 1.2	0.02
Household members (*n* = 431)	4 (2–8)	4 (2–6)	4 (2–8)	0.007
Screen time (hours/day) (*n* = 431)	7 ± 2.8	7.4 ± 3.3	6.9 ± 2.7	0.25
Sitting time (hours/day) (*n* = 431)	6.9 ± 2.5	8 ± 2.8	6.7 ± 2.4	0.002
Step count (pedometer/day) (*n* = 431)	4777 ± 2384	4580 ± 2257	4797 ± 2399	0.65
Junk food consumption (n = 431)	1.8 ± 1.4	2.4 ± 1.6	1.7 ± 1.3	0.006
Breakfast meal				
Consumed	369 (85.6)	39 (95.1)	330 (84.6)	0.07
Skipped	62 (14.4)	2 (4.9)	60 (15.4)	
Lunch meal				
Consumed	358 (83.1)	31 (75.6)	327 (83.8)	0.18
Skipped	73 (16.9)	10 (24.4)	63 (16.2)	
Dinner meal				
Consumed	425 (98.6)	41 (100)	384 (98.5)	0.42
Skipped	6 (1.4)	0	6 (1.5)	
Fruit consumption (portions/day) (*n* = 431)				
Consumed	382 (88.4)	32 (78)	349 (89.5)	0.04
Skipped	50 (11.6)	9 (22)	41 (10.5)	

The BMI of all participants was 18.3 ± 4.2 (kg/m^2^), and BMI SDS was 0.4 ± 1.4. In those with sarcopenic obesity, BMI was 25.5 ± 3.0 (kg/m^2^), and BMI SDS was 2.6 ± 0.4, while in those without sarcopenic obesity, BMI was 17.5 ± 3.6 (kg/m^2^) and the BMI SDS was 0.2 ± 1.3, with a significant difference between the two groups (*p* < 0.05) (Table 3). In those with SO, waist circumference was 78.5 ± 9 cm, waist circumference skinfold thickness was 22.4 ± 7.1, mid-upper arm circumference was 25.6 ± 2.3 cm, mid-upper arm skinfold thickness was 23.8 ± 5.3, and subscapular skinfold thickness was 26.9 ± 5.4. Waist circumference, waist circumference skinfold thickness, mid-upper arm circumference, mid-upper arm skinfold thickness, and subscapular skinfold thickness differed between patients with and without sarcopenic obesity (*p* < 0.05) (Table 3).

When the body fat and muscle composition of the participants are analyzed, the total muscle mass was 29.1 ± 4.6 kg in those with sarcopenic obesity, while it was 22.7 ± 5.6 kg in those without sarcopenic obesity. The fat mass (FM) was 17.5 ± 5.7 kg and 7.4 ± 4.3 kg, respectively. A significant difference was observed between the two groups (*P* < 0.05). The muscle to fat ratio was 1.7 ± 0.3 in those with sarcopenic obesity, and it was 3.5 ± 1 in those without sarcopenic obesity, which shows a significant difference between the two groups (*p* < 0.05) (Table 3).

There was a significant difference between the ratio of handgrip strength (HGS), and it was 0.56 ± 0.11 in those with SO whereas it was 0.72 ± 0.19 in those without SO (*p* < 0.05). Appendicular muscle mass (SMM) was 11.9 ± 2.6 in those with sarcopenic obesity, and the SMM/FM ratio was 0.7 ± 0.1, which was a noteworthy difference between the two groups (*p* < 0.05) (Table 3).

Nutritional information about 193 (44.7%) of the participants was available. The daily caloric intake was 1481 ± 332 kcal/day, with 15.3 ± 4% from protein, 48.2 ± 10.1% from carbohydrates, and 36.4 ± 9.5% from fat. There was no difference in the daily caloric, fat, protein, and carbohydrate content between patients with and without SO (*p* > 0.05). The physical activity scores were 25.7 ± 4.9 in those with sarcopenic obesity, and it was 26.4 ± 5.7 in those without SO. This showed no significant difference between the groups (*p* > 0.05) (Table 3).

### Comparison of lifestyle characteristics between people with and without sarcopenic obesity

The total daily sleep duration of the participants was 9.5 ± 1.16 h. There was an important difference in the total daily sleep duration between those with and without SO (*p* = 0.02). The median number of people living in a household was 4.

Furthermore, the number of people living in a household was significantly different between those with and without SO (*p* = 0.007). The total screen time per day for the entire group was 7 ± 2.8 h, with no difference between the groups (*p* = 0.25). The total sitting time was 6.9 ± 2.5 h/day, with a noteworthy difference between those with and without SO (*p* = 0.002). The daily step count was 4777 ± 2384, with no significant difference between patients with and without SO (p = 0.65).

The junk food consumption for the whole group was 1.8 ± 1.4, and the difference in junk food consumption between those with and without SO was noticeable (*p* = 0.006). Among the participants, 369 (85.6%) had regular breakfast, 358 (83.1%) had regular lunch, and 425 (98.6%) had regular dinner meals. There was no difference in regular breakfast, lunch, or dinner consumption between participants with and without SO (*p* = 0.07, *p* = 0.18, and *p* = 0.42, respectively). Although 382 (88.4%) participants consumed at least one portion of fruit per day, fruit consumption was significantly different between those with and without SO (*p* = 0.04) (Table 3).

### Evaluation of factors affecting sarcopenic obesity development using logistic regression

In the logistic regression analysis examining the factors affecting the development of SO, lack of fruit consumption had an OR of 2.68, 95% CI (1.13–6.31), *p* < 0.05; number of people living in the household had an OR of 0.54, 95% CI (0.35–0.84), *p* < 0.05, increased sitting time had an OR of 1.17, 95% CI (1.02–1.36), *p* < 0.05, and increased junk food consumption had an OR of 1.27, 95% CI (1.03–1.57) *p* < 0.05. Breakfast meal was not found to be significant OR 0.26, 95% CI (0.06–1.18) *p* > 0.05. This has indicated that these factors were effective in the development of SO (Table 4).

**Table 4:** Evaluation of factors affecting sarcopenic obesity development

Risk factor	Univariate analysis unadjusted OR* (95% CI)	*p*	Multivariate analysis adjusted OR* (95% CI)	*p*
Daily fruit consumption				
Consumes	1 (ref)		1 (ref)	
Does not consume	2.39 (1.06–5.36)	0,03	2.68 (1.13–6.31)	0.02
Breakfast meal				
Consumes	1 (ref)		1 (ref)	
Does not consumes	0.28 (0.06–1.19)	0.09	0.26 (0.06–1.18)	0.08
Household members	0,0.8 (0.39–0.86)	0,007	0.54 (0.35–0.84)	0.007
Sitting time (hours)	1.22 (1.07–1.41)	0.004	1.17 (1.02–1.36)	0.02
Junk food consumption				
Consume	1.33 (1.09–1.61)	0.004	1.27 (1.03–1.57)	0.02
Not consume	1 (ref)		1 (ref)	

*OR Odds ratio, a logistic regression analysis was conducted to assess the impact of various factors on the outcome variable. The model included the following independent variables: number of household members, sitting time, junk food consumption, breakfast meal consumption, and fruit consumption

## Discussion

The comprehensive and representative cross-sectional studies, featuring precise body composition parameters and COVID-19 pandemic impact on, presented a unique opportunity to conduct this research. This study, was conducted among children, whose body composition is rapidly developing, especially with the rapid accumulation of muscle and bone during childhood [16, 17]. Compared to adult studies, the result of study also provides important evidence for intervention and COVID-19 pandemic impact on children.

Also, the definition of sarcopenic obesity (SO) varies between studies, and there are a limited number of studies assessing its prevalence and its relation to adverse health outcomes in children [2]. In other words, according to previous studies, the prevalence of SO in girls ranged from 5.6 to 69.7%, and in boys, it ranged from 7.2 to 81.3%. This wide range is due to the different methods used to define SO and the varying cutoff points applied [2]. Although the definition of SO varies between studies, all of the results show that the child SO burden is serious [2]. In this study, the researchers found the prevalence of SO using MFR bioelectric impedance methods to be 8.7% in girls and 10.4% in boys. These findings highlight the crucial importance of monitoring body composition and preventing SO in children, especially during lockdown periods.

Research indicates that during the pandemic, many children experienced increased body weight and altered body composition due to reduced physical activity and changes in eating behaviors. In this study, it was observed that participants with SO had higher BMI SDS (2.6 ± 0.4), waist circumferences 78.5 ± 9 cm, fat mass 17.5 ± 5.7 compared to those without SO. In a review covering the COVID-19 period, 10 studies were evaluated, and it was shown that 59.7% of the participants had an increase in BMI, waist circumference, and body fat mass [18, 19]. During the pandemic period, changes in eating habits, decreased physical activity, and a sedentary lifestyle seem to be effective factors in this situation. It was found that participants with SO also had a decrease in lean mass/fat-free mass and a decrease in their handgrip strength/BMI.

The restrictions of the pandemic, including school closures and lockdowns, led to a marked increase in sedentary behavior among children. This shift in behavior has been linked to an increase in obesity rates among children, with studies reporting a rise in obesity prevalence from 13.7 to 15.4% during pandemic [20].

The findings of this study are consistent with a growing body of research that highlights the impact of the COVID-19 pandemic on increasing rates of childhood and adolescent obesity. The results, which show that 11.8% of participants were overweight and 25.2% were obese, align with reports from Sweden, England, and Turkey, all of which noted a rise in obesity and overweight prevalence during the pandemic [21–26]. This increase is widely attributed to the creation of an obesogenic environment during the pandemic, characterized by school closures, reduced physical activity, and increased sedentary behavior as children spent more time at home [6, 27–30]. Similar to other studies, this research suggests that the disruption of daily routines due to the pandemic, including the shift to remote learning and the restriction of outdoor activities, played a significant role in this trend. The consistent findings across different countries underscore the global nature of this issue, indicating that the pandemic exacerbated existing challenges related to childhood obesity.

In addition, this study aligns with the recommendations of the American National Sleep Foundation, which suggests that children aged 6–10 years should get 9–11 h of sleep per night [31]. However, the findings of this study reveal that during the pandemic, while the average sleep duration for children was around 10 h, there were notable shifts in their sleep routines which were characterized with children going to bed later and waking up later. This change in sleep patterns is consistent with other studies that have documented how the uncertainty and anxiety brought about by the pandemic, coupled with the shift to remote learning, disrupted children’s regular sleep schedules. These disruptions, in turn, have had notable implications for children’s mental and physical health [32–35]. The findings of the study also introduce a new consideration as the specific context of the pandemic may have intensified these relationships. Unlike typical circumstances, the pandemic’s unique conditions—such as prolonged periods of confinement and stress—may have exacerbated the effects of sleep disruption on obesity. Moreover, this current study identified a difference in sleep duration between children with SO which is shorter sleep durations observed in children with SO. This finding is consistent with research in adults that links short sleep duration to obesity [36], since shorter sleep may lead to disruptions in appetite-regulating hormones and reduced physical activity due to fatigue.

Furthermore, the findings in this study are in line with several other studies that have documented an increase in screen time and sitting time among children during the COVID-19 pandemic. One of the main reasons for the increased sitting time and screen time was pandemic-related restrictions. With home confinement, children mostly spent their time at home. Therefore, the screen time increased in order to follow the online education and courses. With the COVID-19 pandemic, the new normal was integrated into our lives, and one of the biggest changes occurred in the family life and children education system. During this period, the distance education model was applied. Similar to research conducted Germany, Italy, and Asia, this study observed that children spent more time on electronic devices and engaged in more sedentary behaviors due to pandemic related restrictions [28, 37, 38]. In this study, the screen time was 6.9 ± 2.8 h, with 6.8 ± 2.5 h sitting daily. Sitting time increased the risk of SO (OR 1.17, *p* = 0.02), highlighting the need to address sedentary behavior [39, 40]. Another crucial challenge during the pandemic was limited outdoor access. In short, it needs to be noted that sitting time is linked to SO through its detrimental effects on muscle mass and fat distribution. Prolonged sitting leads to muscle atrophy and increased fat accumulation, both of which are key components of SO.

Studies indicate that children with obesity are at a heightened risk of developing sarcopenic obesity, especially if they engage in limited physical activity. For instance, a comprehensive review highlighted that physical inactivity, often exacerbated by modern sedentary lifestyles, contributes significantly to the development of sarcopenic obesity in younger populations [41]. In other words, each additional 30 min per day of sedentary time was marginally associated with an increased risk of SO, independent of moderate-to-vigorous physical activity [39, 42]. In current study, the mean physical activity (PA) was 26.4 ± 5.6, with no SO group difference, suggesting that prolonged sitting may increase the risk of SO, which was independent of PA.

As far as physical activity is concerned, it needs to be considered that it offers numerous benefits for children’s health. For children aged 5–17 years, it is recommended to engage in at least 60 min of moderate-intensity physical activity daily, with at least 3 days a week focused on strengthening muscles and bones. Additionally, several hours of light physical activity (such as walking or playing) are also recommended each day [43, 44].

During the pandemic, several studies found that children and adolescents were getting 45–91 min less physical activity each day [45]. A study involving 1472 Canadian children showed that only a small number of them managed to get the recommended 60 min of moderate-intensity physical activity daily [46]. Similarly, research from South Korea, Croatia, and Turkey highlighted a noticeable drop in children’s sports and play activities during the pandemic, which was observed especially among those living in urban areas [47–49].

While some studies reported increases in activity, the decrease of steps was likely due to the closure of physical activity spaces [40, 50]. Considering the 12,000 steps/day optimal level, it was observed that the activity declined during lockdowns [51, 52]. In this study, it was found that children took an average of 4392 steps per day and had a physical activity score of 26, which suggests they can be considered moderately active. However, no significant difference was observed in physical activity scores between the sarcopenic obese and non-obese groups. This may be because the level of physical activity measured in the study was not sufficient to reduce the risk of SO. It also indicates that the children in this study did not fully meet the recommended guideline of at least 60 min of moderate to vigorous physical activity per day. As a result, this can lead to a decrease in muscle mass and strength, which can increase the risk of sarcopenia and SO.

Another important finding of this study is the difference in the number of household members between the sarcopenic obese and non-sarcopenic obese groups, with results showing that an increase in household members reduces the risk of sarcopenic obesity by 0.54 times. Although the literature does not explicitly discuss the impact of household members on sarcopenic obesity, it is plausible that the number of household members could influence lifestyle choices, including physical activity levels and sedentary behavior. For instance, individuals living in larger households may engage in more physical activities due to social facilitation or may have less sedentary time due to household responsibilities.

The COVID-19 pandemic, along with measures like lockdowns, social isolation, and school closures, not only created environments conducive to obesity but also indirectly influenced dietary patterns [53]. In this study, the authors found out that 67% of children did not consume enough fruit and one in four consumed more than three junk food items per day. By the same token, previous studies have shown that during the pandemic, regular meal patterns and fruit and vegetable consumption decreased while the intake of sweets and snacks increased among children [28, 54, 55]. Therefore, the increased consumption of unhealthy, high-calorie foods during the COVID-19 pandemic may have played a role in the development of unhealthy body composition changes, including increased fat mass and decreased muscle mass, leading to sarcopenic obesity in children and adolescents.

Moreover, social distancing measures may have also impacted agricultural production, transportation, and the availability of nutritious, fresh, and affordable foods, leading families to opt for lower-nutrient alternatives. Studies have shown that consuming high-fiber fruits and vegetables can reduce skinfold thickness and fat mass ratios [56, 57]. Particularly in adult studies, there is evidence linking antioxidant-rich foods and fruit intake to a lower risk of sarcopenic obesity. Furthermore, previous research has found that older adults who frequently consume fruits and vegetables have a 68% lower risk of developing sarcopenia [58, 59]. In this study, the researchers also determined a connection between fruit consumption and the development of sarcopenic obesity. The antioxidants like carotene and vitamin C found in fruits and vegetables may help reduce oxidative stress in skeletal muscles, which potentially lowers the risk of sarcopenia.

The strength of this study is that it is a comprehensive study that provides a detailed examination of the impact of physical activity, dietary habits, sleep patterns, and media use on sarcopenic obesity in children during the COVID-19 pandemic. It included a large participant group, and it is a prospective, cross-sectional study conducted with 431 child participants.

The limitation of this study arises from dietary data and physical activity levels relied on self-reported formats, which may be subject to reporting biases. As it only included children aged 6–10 years, the generalizability may be limited.

## Conclusion

Sarcopenic obesity (SO) is an emerging concern in pediatric populations, especially during periods of reduced physical activity and altered lifestyles, such as the COVID-19 pandemic. This study highlights the significant impact of the pandemic on children’s body composition, with an increased prevalence of obesity and SO among those aged 6–10 years. Factors such as decreased physical activity, increased screen and sitting time, poor dietary habits, and changes in sleep patterns have been identified as contributing to the development of SO in children. The findings highlight the critical need for targeted interventions that promote healthy eating habits, regular physical activity, and adequate sleep to mitigate the risks associated with SO. Efforts should focus on creating supportive environments that encourage active lifestyles and reduce sedentary behaviors, particularly during times of crisis that disrupt normal routines. Future research should aim to establish standardized diagnostic criteria for SO in children and explore effective prevention and management strategies to address this growing health concern.

## Data Availability

No datasets were generated or analysed during the current study.

## Abbreviations

SO: Sarcopenic obesity
PAQ-C: Physical activity questionnaire for older Children
TUBER: Turkish dietary guidelines
BEBIS 8.2: Nutritional Information Systems Package Program
MUAC: Mid-upper arm circumferences
WHO: The World Health Organization
BMI: Body mass index
MFR: Muscle-fat ratio
Appendicular muscle mass: Sum of the muscle mass of the right arm, left arm, right leg, and left leg
TOÇBI: The monitoring of growth in school age children in Turkey
PA: Physical activity
BIA: Bioelectric impedance
KNHANES: Korean National Health and Nutrition Examination Survey
